# Scarlet Fever

**Published:** 1887-09-03

**Authors:** 


### THE DOCTOR'S PULPIT.

### SCARLET FEVER.

At the present time London is threatened with an epidemic of scarlet fever. This is one of those pestiferous diseases which make a certain period of childhood both anxious and dangerous. It is by no means so fatal as small-pox used to be before the discovery of the prophylactic power of vaccination; but if any modern Jenner could discover a preventive so easy of application and so generally efficacious as that used against small-pox, he would be one of the greatest benefactors to his kind the recent centuries have seen. Nothing gives a poorer idea of the general mental capacity of mankind than the quasi-indifference of the public to the name and fame of Jenner. With the instructed his immortality is assured. But then his discovery was not a salvation merely for the instructed: it was, like the rain, or the dew, or the sunshine, a free gift bestowed upon all men. The Romans and the French have raised triumphal arches to the memory of those who have been most famous in the shedding of rivers of blood; the English have raised no arches at all; but if they should by any possibility be seized with an attack of enthusiasm, grateful or otherwise, they cannot do better than expend it in the raising of a triumphal arch to Jenner, magnificent enough to overshadow the most splendid in Paris or Rome.

The germ theory of disease now holds the field; and scarlet fever is said to be caused by germs. Germs are little living bodies, that under favourable circumstances thrive and propagate their kind with extraordinary rapidity, to the serious distress and danger of the person in whom the process is taking place. Just as there is great variety of form, character, and life-history in those animal and vegetable organisms which can be seen without the microscope all the world over, so is there, in what may be called the microscopic world, an almost infinite variety of shape, habit, and conduct. A germ is a germ, it may be said. True, and an animal is an animal, but it may be an elephant or a tomtit; a flea or a whale. So with germs: one may be small, and another infinitely smaller: one may be straight and another twisted, and so on. But like animals and plants they have certain characteristics in common which justify their passing by the common name of "germs." What does it really matter whether scarlet fever is caused by germs or not? A most ignorant question, and one which it would be very discouraging to hear from a doctor's lips. It matters as a question of knowledge; but it matters also as a question of treatment. If scarlet fever be caused by germs, then by keeping every non-infected person away from contact with the germs, the disease may be promptly prevented from spreading. Not only so, but if the germs can only develop and propagate where they find a suitable habitat and adequate nutrition, it may be possible, as in the case of small-pox, so to alter the conditions of the habitat and to exhaust the nutrition that the disease may almost be expelled from the world entirely.

Mr. Winter Blyth, Medical Officer of Health for Marylebone, was under the impression some time ago, and possibly still is, that he had made an important discovery with regard to scarlet fever. He thought he had found out that it might be transmitted from the cow by means of the milk. There was an outbreak of scarlet fever in his district, and it was found that all or most of the houses in which the disease occurred were supplied with milk from one particular dairy which was situated at Hendon. At the dairy several cows were suffering from an affection of the udder and teats. Was there any connection between this affection and the outbreak of scarlet fever? Mr. Blyth thought there might be. Dr. Klein, an accomplished cultivator of germs, was called in. He obtained some of the scabs and other products of the disease from the udders of the affected cattle, and, after making cultivations and trying them on animals, he came to the conclusion that at any rate there was a degree of probability, almost amounting to certainty, that the scarlet fever outbreak had been caused by the presence in the milk of the products of the disease in the cows at Hendon. Mr. Power, who had taken part in the investigation throughout, concurred in this view. Professor Axe, the veterinary surgeon, did not concur. Dr. Thin, a man of critical acumen and good reasoning powers, submitted the whole story, with other facts ascertained by himself, to judicial investigation, and has recently given his reasoned opinion thereupon. His verdict is the Scotch one of "Not proven"; and there is not the slightest doubt that this pronouncement is warranted by the whole of the facts. The Hendon case must be considered as still *sub judice;* and so far as our scientific knowledge of scarlet fever is concerned we are exactly where we were before, excepting that the researches of Mr. Power, Dr. Klein, Dr. Allan Jamieson, and Mr. Eddington decidedly favour the "germ" theory of its origin.

Whatever be the actual truth about "germs," it is admitted on all hands that scarlet fever is a contagious disease, and that its power of infection by contact is very marked. It is probable, though not certain, that children kept at a sufficient distance from every source of infection would never have scarlet fever at all There is a delusion abroad that at one time or other of their lives they *must* have it; and many people allow a boy or girl who already has the disease to associate with the rest of the family. This is folly of the maddest kind, and would be criminal if it were not done ignorantly. Three or four years ago scarlet fever broke out in a family in London. The doctor, with culpable ignorance, did not insist upon isolation, but encouraged the idea that as the remaining children were almost sure to catch the disease sooner or later, it would be better for them to have it sooner rather than later. The fever patient was therefore allowed to run about with the other children, with the result that, out of a family of four, three took it and were dead in six weeks.

It is a point of importance to remember that the disease seems to be much more feebly infectious in its early stages than later on. There are some who assert that it is not infectious at all until "peeling" has commenced. That is an assertion which cannot be proved, and should never be relied upon in practice. The rule should be this: During an epidemic of scarlet fever, as soon as a patient, especially a child, shows any signs of illness of any kind, isolate it immediately. If the case proves to be not one of scarlet fever, so much the better. No harm has been done by the few days' isolation, and it may be that dangerous risks have been avoided. There is a period of what is called "incubation" of the disease, in which it is impossible for the most skilful doctor to say what is the nature of the developing attack. This period may last from four to six or eight days, or possibly even longer. Then is the time, if there be any scarlet fever in the neighbourhood, to make isolation as complete as it can possibly be made. A separate room on a separate floor, well shut off by sheets soaked in some disinfectant; a separate attendant, with separate passages, where possible, to the domestic parts of the house—all these should be insisted upon. Isolation cannot be too complete. To do it partially is to put out the fire in a burning house in every room but one, and in that to leave it raging unchecked. It would have been just as well to have left the fire-engine and the firemen at the station in peace. Here, if anywhere, thoroughness must be the order of the day. Whilst on the subject of infection, it may be well to complete it by saying that the poison of scarlet fever is most active during the "peeling" period, and afterwards lingers about for a great and quite unknown length of time; and that not only on the person of the patient, but also about the clothing, the bedding, the bed-curtains (which, by the way, should always be removed at the very commencement of the attack), about the wall-papers, ceilings, floors, and indeed every place where it can possibly obtain a footing. The patient cannot be considered fit to mix with others at all in less than six or eight weeks; and then only after thorough ablution in warm water mixed with a disinfectant. The clothing, bedding, etc., should all be burnt or disinfected with the utmost care. The room should undergo the same complete process, and before being again occupied should be whitewashed with lime, repapered, and the floors energetically scrubbed with hot water and disinfectants of the strongest kind. The master or mistress of the house should personally see that all this is done in the most effectual way. The importance of it cannot be exaggerated.

After the fever stage is over the danger to the patient is not past. For two or three weeks there is marked risk of inflammation of the kidneys, an affection which in most cases is much more dangerous than the fever. The slightest chill may produce this; and a case which has been going on most favourably may die from getting out of bed too early. Every person attacked with scarlet fever, even in its mildest form, should be kept in bed or in a room uniformly warmed, for three weeks; in most cases for a month, if absolute safety is to be secured. Rheumatism and other effects of debility are also frequent after this most insidious disease.

With regard to treatment, this must of course be carried out under the instructions of the medical attendant. Many people deny fever patients a drink of cold water, from the mistaken impression that it is in some way injurious; and they pile on the bedclothes mountains high. Oh for a little sense! Filter and boil the water by all means, and then when it is cooled let the fevered tongue and mouth have it freely and frequently. Put in a little ice too: a piece of ice in the mouth is often most pleasant and beneficial. Do not on any account pile on too many bedclothes! Think how you would feel if you were blazing hot, and some misguided friend were to heap mountains of blankets and counterpanes upon you! Of course care must be taken to avoid the opposite extreme. There is nothing more dangerous than a chill in scarlet fever.

It should always be remembered that in this, as in most other fevers, nursing is nine-tenths of the battle. The doctor will do his best to lower the temperature, to order medicines for the throat and mouth, and generally to guide the attendant in charge from day to day. But when he is not there, the regular feeding, the soothing drink, the turning at the right time, the opening or closing of the window, the pulling down or up of the blind, the quietness, the cheerfulness, the hope,—these are the things which none but the nurse can attend to, and these may make the difference between recovery and death. Scarlet fever is eminently one of those diseases which cannot be "cured" or cut short. It must and will run through its course, either mildly or severely. But whether the patient shall be made as comfortable as possible whilst it lasts; or whether he shall recover or die, depends to a very large extent on the care and intelligence displayed by the attendant nurse.

*(To be continued.)*

